# Acute kidney injury is common, parallels organ dysfunction or failure, and carries appreciable mortality in patients with major burns: a prospective exploratory cohort study

**DOI:** 10.1186/cc7032

**Published:** 2008-10-10

**Authors:** I Steinvall, Z Bak, F Sjoberg

**Affiliations:** 1 The Burn Unit, Department of Hand and Plastic Surgery, Linköping University Hospital, Garnisonsvägen, Linköping, 58185, Sweden; 2 Department of Clinical and Experimental Medicine, Faculty of Health Sciences, Linköping University Hospital, Garnisonsvägen, Linköping, 58185, Sweden; 3 Department of Anesthesia and Intensive Care, Linköping University Hospital, Garnisonsvägen, Linköping, 58185, Sweden

## Abstract

**Introduction:**

The purpose of this study was to determine the incidence, time course, and outcome of acute kidney injury after major burns and to evaluate the impact of possible predisposing factors (age, gender, and depth and extent of injury) and the relation to other dysfunctioning organs and sepsis.

**Method:**

We performed an explorative cohort study on patients with a TBSA% (percentage burned of total body surface area) of 20% or more who were admitted to a national burn centre. Acute kidney injury was classified according to the international consensus classification of RIFLE (Risk, Injury, Failure, Loss of kidney function, and End-stage kidney disease). Prospectively collected clinical and laboratory data were used for assessing organ dysfunction, systemic inflammatory response, and sepsis.

**Results:**

The incidence of acute kidney injury among major burns was 0.11 per 100,000 people per year. Of 127 patients, 31 (24%) developed acute kidney injury (12% Risk, 8% Injury, and 5% Failure). Mean age was 40.6 years (95% confidence interval [CI] 36.7 to 44.5), TBSA% was 38.6% (95% CI 35.5% to 41.6%), and 25% were women. Mortality was 14% and increased with increasing RIFLE class (7% normal, 13% Risk, 40% Injury, and 83% Failure). Renal dysfunction occurred within 7 days in 55% of the patients and recovered among all survivors. Age, TBSA%, and extent of full thickness burns were higher among the patients who developed acute kidney injury. Pulmonary dysfunction and systemic inflammatory response syndrome were present in all of the patients with acute kidney injury and developed before the acute kidney injury. Sepsis was a possible aggravating factor in acute kidney injury in 48%. Extensive deep burns (25% or more full thickness burn) increased the risk for developing acute kidney injury early (risk ratio 2.25).

**Conclusions:**

Acute kidney injury is common, develops soon after the burn, and parallels other dysfunctioning organs. Although acute kidney injury recovered in all survivors, in higher acute kidney injury groups, together with cardiovascular dysfunction, it correlated with mortality.

## Introduction

Renal failure is a feared complication of critical illness and is also often an early sign of multiple organ dysfunction, which complicates the care of critically ill patients [1-4]. In modern burn care, in which most patients now survive early resuscitation, multiple organ failure is the most common cause of death. In the largest database of patients with burn injuries, the American Burn Association burn registry, records of the cause of mortality indicate that 49% of the non-survivors died of organ failure [5]. The incidence of acute kidney injury (AKI) among burned patients varied from less than 1% to 36%, depending on the population studied and the criteria used for classification (Table 1). Another shortcoming was that most studies were carried out retrospectively. Of the studies that claimed to collect data prospectively, not all measures of organ failure were collected according to a true prospective protocol [6,7]. It is therefore obvious that there is a risk that organ dysfunction may have been overlooked or missed. The mortality among burned patients who developed AKI was between 28% and 100% and was 50% to 100% among those who were treated with renal replacement therapy. The reported incidence of renal replacement therapy varied from 0.7% to 14.6% (Table 1) [6-22].

**Table 1:** Incidence, mortality, and criteria for acute kidney injury in patients with burns

Reference	Year	Years of study; population	AKI	AKI mortality	Criterion of AKI
Davies, *et al*. [8]	1979	1958–1979; >1,064 patients admitted	28 (<2.6%)	24 (86%)	Renal replacement therapy
Davies, *et al*. [9]	1994	1991; 18 burn units	15 (<1%)	12 (80%)	Renal replacement therapy
Leblanc, *et al*. [10]	1997	1987–1994; 970 patients admitted	16 (1.6%)	13 (82%)	Renal replacement therapy
Holm, *et al*. [11]	1999	1994–1998; 328 patients, 34% TBSA%	48 (15%)	41 (85%)	Renal replacement therapy
Tremblay, *et al*. [12]	2000	1995–1998; 353 patients admitted	12 (3.4%)	6 (50%)	Renal replacement therapy
Schiavon, *et al*. [13]	1988	1988; 20 patients, 44% TBSA%	4 (20%)	4 (100%)	Serum creatinine raised >133 μmol/L above value on admission
			0		Renal replacement therapy
Saffle, *et al*. [7]	1993	1987–1990; 529 patients, 16% TBSA%	50 (10%)	23 (46%)	Thermal Injury Organ Failure Score (moderate: serum creatinine >222 μmol/L)
			4 (0.8%)	4 (100%)	Renal replacement therapy
Sheridan, *et al*. [14]	1998	1989–1994; 56 patients who died	37 (68%)	-	Serum BUN ≥100 and creatinine ≥3.5 or urine output ≤500 mL/day
Jeschke, *et al*. [15]	1998	1966–1997; 5,000 children admitted	60 (1.2%)	44 (73%)	Oliguria (<0.5 mL/kg per hour for >36 hours), serum urea nitrogen/creatinine ratio <20, serum creatinine >177 μmol/L
			34 (0.7)	28 (82%)	Renal replacement therapy
Chrysopoulo, *et al*. [16]	1999	1981–1998; 1,404 patients, TBSA% >30%	76 (5.4%)	67 (88%)	Three of these four: oliguria (<350 mL/36 hours), BUN/creatinine ratio <20, serum creatinine >177 μmol/L, and dialysis
			67 (4.8%)	61 (91%)	Renal replacement therapy
Kim, *et al*. [17]	2003	2000; 147 patients, 60% TBSA%	28 (19%)	28 (100%)	Serum creatinine >177 μmol/L
			3 (2.0%)	3 (100%)	Renal replacement therapy
Mustonen and Vuola [22]	2008	1988–2001; 238 patients, 31% TBSA%	93 (39.1%)	41 (44%)	Serum creatinine >120 μmol/L
			32 (13%)	20 (62%)	Renal replacement therapy
Cumming, *et al*. [6]	2001	1998–1999; 85 patients, 30% TBSA%	3 (3.5%)		MODS (3–4: serum creatinine >350 μmol/L)
Cooper, *et al*. [18]	2006	1999–2001; 42 patients, 35% TBSA%	3 (7.1%)		MODS (3–4: serum creatinine >350 μmol/L) or oliguria
Coca, *et al*. [19]	2007	1998–2003; 304 patients, 27% TBSA%	81 (27%)	23 (28%)	RIFLE
				(73%)	Renal replacement therapy
Lopes, *et al*. [20]	2007	2004–2006; 126 patients, 24% TBSA%	45 (36%)	21 (47%)	RIFLE
			11 (8.7%)		Renal replacement therapy

The table shows number of patients who had acute kidney injury (AKI) according to the criteria in the rightmost column; the percentage is the incidence of AKI among the study population. AKI mortality is the number of patients who died among those with AKI, with the percentage referring to mortality among the AKI patients. When available, incidence and outcome of renal replacement therapy are shown in the table, together with the result from the primary AKI criteria. Percentage burned of total body surface area (TBSA%) is the mean of the study group. When a TBSA% limit for inclusion was reported instead, it is shown in this table as 'TBSA% >%'. BUN, blood urea nitrogen; MODS, Multiple Organ Dysfunction Score; RIFLE, the increasing severity classes Risk, Injury, and Failure and the two outcome classes Loss, and End-stage renal disease.

It is evident therefore that the definitions, protocols, and collection of data vary considerably among different studies, which makes it difficult to compare results. For the present investigation, we chose to use the RIFLE (Risk, Injury, Failure, Loss of kidney function, and End-stage kidney disease) classification, which was developed recently by the Acute Dialysis Quality Initiative Group and published as a consensus definition of acute renal failure in critical care [23]. We set up the following hypotheses about the present study: first, AKI is common and develops soon after a major burn. Second, it is affected by factors that are described as important for the development of multiple organ dysfunction or failure in patients with burns such as age [6,7,24], percentage burned of total body surface area (TBSA%) [6,7,24], and sepsis [11,12,15,16,19]. As AKI develops together with failure of other organs [7,11,14] and outcome depends on the number and degree of failing organs [6,7,24], assessment of organ failures was made in parallel with the sequential organ failure assessment (SOFA) [25], which is well documented and validated [26-28].

## Materials and methods

**Figure 1:**
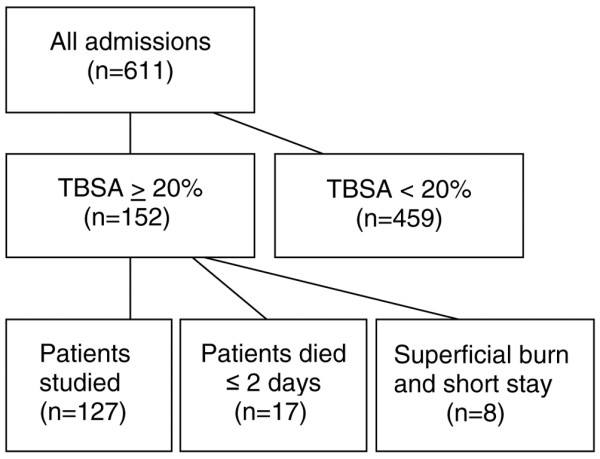
Algorithm showing selection of patients. TBSA, percentage burned of total body surface area.

The burn centre serves 3.3 million inhabitants for referral of patients who require specialist burn care (major burns) from the southern part of Sweden. Consecutive patients with a TBSA% of 20% or more, who were admitted between 1997 and 2005 (8.5 years), were studied. Clinical and laboratory data, collected according to a preset protocol, were recorded during the study period. Patients who died within the first 2 days, including those from whom treatment was withheld or withdrawn early, were excluded. Patients with superficial burns that did not require operation and whose time in hospital was short (1 to 7 days) were also excluded (Figure 1). The local ethics committee at Linköping University Hospital waived the need for their approval for descriptive and explorative studies that do not include any procedures that are not considered as ordinary burn care.

### Treatment of burns and supportive intensive care

Ringer's acetate was used for fluid resuscitation in volumes according to the Parkland formula (4 mL/kg body weight [BW]/TBSA%) [29-31], with adjustments for individual variations in hemodynamic variables, aiming at least for a mean arterial pressure of 70 mm Hg and a urine output of 1 mL/kg BW per hour. When fluid volume substitution alone was insufficient to maintain central hemodynamics, adrenergic drugs (dobutamine and norepinephrine) were used. Renal replacement therapy was considered when the plasma creatinine concentration exceeded 300 μmol/L, together with oliguria or anuria.

Excision and grafting operations were done within 24 to 48 hours. Patients who did not seem to have deep burns at primary examination were re-evaluated daily and operated on if full thickness burns (FTBs) were identified. Wounds were covered by autologous grafts when available or, in extensive burns, either by heterologous grafts for temporary cover or (in special cases) by cultured keratinocytes. Operations were repeated when donor sites again became available. Silver sulfadiazine (Flamazine^®^; Smith & Nephew, Hull, UK) was applied to both grafted and non-grafted wounds. Infection control was managed in collaboration with the university hospital infection specialists.

Ventilation was pressure-controlled (Siemens 900 C or Siemens 300 A; Siemens, Solna, Sweden) with tidal volumes of 6 to 8 mL/kg BW, a positive end-expiratory pressure of at least 5 cm H_2_O, and aiming at low ventilatory plateau pressures (of less than 35 cm H_2_O) [32]. Nutrition was provided enterally from day 1, pain was controlled by continuous infusions of opioids, and sedation was carried out by infusion of benzodiazepines.

### Classification of acute kidney injury

AKI was classified by a dynamic classification scheme with three levels for acute renal dysfunction: Risk, Injury, and Failure and two clinical outcomes, Loss of kidney function and End-stage kidney disease (RIFLE) [23]. It is based on how the plasma creatinine concentration is increased compared with the baseline value of the individual patient, reduced urinary output, and the need for renal replacement therapy. The earliest available plasma creatinine concentration measurement was used as the baseline. Plasma creatinine concentrations from the first week, and thereafter the highest value weekly, were used for RIFLE classification and assessment of renal recovery.

### Classification of organ dysfunction

SOFA score was recorded at admission and at least three times a week. SOFA score is based on the assessment of six organ dimensions: (a) renal: plasma creatinine concentration or urine output, (b) respiratory: arterial partial pressure of oxygen/fraction of inspired oxygen (PaO_2_/FiO_2_) ratio, (c) cardiovascular: hypotension or need for adrenergic agents, (d) coagulation: platelet count, and (e) hepatic: plasma bilirubin concentration. The neurological part of SOFA (f) was left out because of the difficulties in assessing the Glasgow coma score in sedated patients. Maximum SOFA is the maximum score value from each organ score, regardless of date [27]. For this study, multiple organ failure was defined as 3 to 4 score points in two or more organ dimensions of the SOFA score [26]. Blood samples were drawn at the time of admission and at least three times a week in accordance with the Burn Unit protocol for major burns. Admission values were used to compare baseline values among groups; the worst value of each patient during the first week was used to analyse factors of importance for AKI. The worst overall value was used to analyse factors of importance for mortality, and the worst value of each patient each week was used to calculate the maximum SOFA score and the descriptive figures of the time course. Laboratory variables were analysed by routine methods and data were stored in the countywide database of the laboratory. Sepsis and systemic inflammatory response syndrome (SIRS) were classified according to the American College of Chest Physicians/Society of Critical Care Medicine Consensus Conference [33].

### Additional data acquisition

All patients were recorded prospectively in the Linköping Burn Unit Database. At admission, extent (TBSA%) and depth (FTB%) of injury were recorded together with patient characteristics such as age and gender [34]. Daily recordings of care and treatment included variables such as requirement for dialysis, mechanical ventilation, and adrenergic agents. Data regarding the giving of nephrotoxic antibiotics (vancomycin, aminoglycosides, and amphotericin B) and exposure to intravenous contrast (computed tomography [CT] scans) were extracted from medication charts.

### Statistics

Data were analysed with STATISTICA 7 (StatSoft, Inc., Tulsa, OK, USA) and presented as mean and 95% confidence interval (CI). The differences in baseline characteristics and outcome among patients with and without AKI and the differences in mean values between those who developed AKI early and late were analysed using Student *t *test for continuous data and contingency tables with Pearson chi-square test for categorical variables. Analysis of covariance was used adjusting laboratory data for age and TBSA%. The Tukey unequal N HSD (honest significant difference test for unequal sample sizes) was used as a *post hoc *test. The difference in progress time was analysed by using Student *t *test for dependent samples. One-way analysis of variance, for continuous data, and contingency tables with Pearson chi-square test for categorical variables were used to analyse the differences in baseline characteristics and outcome among the patients who developed AKI, grouped in RIFLE classes. Continuous variables were arbitrarily categorised when exploring risk factors for the development of early and late AKI with odds ratios: cutoff age of 60 years, FTB% of 25%, TBSA% of 50%, and reaching the level for Risk within the first 7 days for early AKI.

## Results

### Incidence

The incidence of AKI among major burns was 0.11 per 100,000 people per year during the study period. (See selection of patients in Figure 1.) For the majority (14 of 17) of the excluded patients who died within 2 days, active burn care was withheld or withdrawn because of extensive and deep burns, and 8 of the 14 patients were more than 70 years old. They were older (71.1 years, 95% CI 63.8 to 78.5) and had more extensive burns (58.6% TBSA%, 95% CI 46.4% to 70.8%) than the 127 patients in the study group (*P *<0.001). Three patients had renal failure before active treatment was withdrawn, but no renal replacement therapy was started. Eight patients with superficial burns (25.1% TBSA%, 95% CI 20.3% to 29.9%) that did not require operation and who were inpatients for only a short period (5.3 days, 95% CI 0.68 to 9.82) were also excluded.

A total of 127 patients remained in this study (Table 2), of whom 24% developed AKI (11.8% Risk, 7.9% Injury, and 4.7% Failure) and 3% required renal replacement therapy (Table 3). Overall mortality was 14%. Twenty-nine of the 31 patients who developed AKI had flame burns. One of the two remaining patients had an electrical burn, and one had a chemical hot scald burn (industrial); both were classified as Risk. Seven of the 31 patients (1 classified as Risk, 2 as Injury, and 4 as Failure) had previous histories of hypertension, but none had a documented history of renal dysfunction. One of the patients who was classified as Risk had a previous history of taking lithium. No others had histories of diagnoses affecting the kidney before the burn.

**Table 2:** Characteristics, baseline, and outcome of patients studied who were classified by RIFLE

	No AKI (n = 96)	AKI (n = 31)	*P *value	Adjusted
Age, years	35.9 (31.8 to 40.1)	55.1 (47.4 to 62.7)	<0.001	
Total body surface area, percentage burned	35.8 (33.0 to 38.5)	47.2 (38.3 to 56.1)	0.001	
Full thickness burns, percentage	13.6 (10.9 to 16.4)	32.0 (24.0 to 40.0)	<0.001	
Gender, female/male	22/74	10/21	0.30	
Mortality	7 (7.3%)	11 (35.5%)	<0.001	
Multiple organ failure	3 (3.1%)	24 (77.4%)	<0.001	
Mechanical ventilation	51 (53.1%)	30 (96.8%)	<0.001	
Length of stay for survivors, days	39.9 (32.5 to 47.3)	67.3 (46.0 to 88.6)	0.004	
Baseline laboratory variables				
Plasma creatinine, μmol/L	81.3 (76.4 to 86.1)	82.3 (72.0 to 92.5)	0.85	0.87
Platelet count, × 10^9^/L	238 (218 to 259)	278 (231 to 326)	0.08	0.14
Plasma bilirubin, μmol/L	18.9 (15.5 to 22.3)	24.0 (17.3 to 30.6)	0.13	0.21
Worst laboratory value during the first week				
Lowest platelet count, × 10^9^/L	120 (106 to 133)	68 (48 to 87)	<0.001	0.001
Plasma bilirubin, μmol/L	19.9 (16.7 to 23.0)	37.4 (26.1 to 48.6)	<0.001	0.001

Data are mean (95% confidence interval) or number (percentage). Acute kidney injury (AKI) is classified by RIFLE (Risk, Injury, Failure, Loss of kidney function, and End-stage kidney disease). Multiple organ failure is 3 to 4 sequential organ failure assessment score points in more than one organ dimension. Worst laboratory value is the highest value for bilirubin and the lowest value for platelet count. We used contingency table Pearson chi-square test for categorical variables, Student *t *test for continuous data, and analysis of covariance (with *P *value from *post hoc *analysis between AKI and no AKI) to adjust for age and for percentage burned of total body surface area.

**Table 3:** Characteristics, baseline, and outcome of the patients who developed acute kidney injury classified by RIFLE (n = 31)

	Risk (n = 15)	Injury (n = 10)	Failure (n = 6)	*P *value
Age, years	47.7 (36.1 to 59.2)	56.9 (42.7 to 71.1)	70.5 (55.1 to 85.9)	0.07
Total body surface area, percentage burned	45.6 (32.5 to 58.7)	56.5 (37.1 to 75.9)	35.8 (17.8 to 53.9)	0.25
Full thickness burns, percentage	32.4 (19.9 to 44.8)	36.0 (17.2 to 54.9)	24.3 (14.4 to 34.3)	0.60
Gender, female/male	6/9	2/8	2/4	0.58
Mortality	2	4	5	0.01
Dialysis	-	-	4	
Recovery	13	5^a^	2^b^	0.04
Multiple organ failure	9	9	6	0.07
Lowest mean arterial pressure, mm Hg	56.0 (53.2 to 58.8)	62.2 (56.1 to 68.3)	57.2 (52.4 to 61.9)	0.06
Adrenergic drugs on days 1–3^c^	11	5	5	0.31
Mechanical ventilation	15	9	6	0.34^d^
Length of stay for survivors, days	69.2 (40.0 to 98.3)	66.8 (17.2 to 116.4)	46	0.90

Data are mean (95% confidence interval) or number of patients. Acute kidney injury is classified by RIFLE (Risk, Injury, Failure, Loss of kidney function, and End-stage kidney disease). Multiple organ failure is 3 to 4 sequential organ failure assessment score points in more than one organ dimension. The lowest recorded mean arterial pressure from days 1 to 3 was used. ^a^One patient was transferred to another hospital before recovery. ^b^The patient who survived Failure was partially normalised after 7 weeks, and one patient whose recovery was complete after 9 weeks died after 16 weeks. ^c^Number of patients who required adrenergic drugs during days 1 to 3. We used contingency table Pearson chi-square test for categorical variables (^d^three of the six expected values were less than 1) and one-way analysis of variance for continuous data.

Half of the patients who developed AKI (55%, 17 of 31) reached the level for Risk within the first 7 days, and 81% (25 of 31) within 14 days. The progress time from Risk to maximum RIFLE class was 5.2 days (95% CI 2.0 to 8.5) among the 16 patients who reached Injury and Failure, whereas the time from baseline to Risk was 9.4 days (95% CI 5.9 to 13.0) (*P *= 0.095). Early AKI was arbitrarily defined as when creatinine reached the level for Risk within the first 7 days, late AKI between days 8 and 60 (Table 4). We found a more than twofold higher risk for younger patients (risk ratio 2.35) and for patients with extensive deep burns (risk ratio 2.25) to develop AKI early (Table 5).

**Table 4:** Early and late acute kidney injury: characteristics, multiple organ failure, and sepsis

	Early AKI (n = 17)	Late AKI (n = 14)	*P *value
Age, years	48.9 (39.7 to 58.1)	62.6 (49.7 to 75.5)	0.07
Total body surface area, percentage burned	53.3 (41.0 to 65.6)	39.8 (26.4 to 53.2)	0.13
Full thickness burns, percentage	39.4 (28.1 to 50.7)	23.0 (12.2 to 33.8)	0.04
Multiple organ failure	14	10	0.47
Sepsis	15	12	0.83
Lowest value of MAP for days 1–3, mm Hg	57.5 (54.9 to 60.2)	59.1 (54.3 to 63.9)	0.53
Plasma myoglobin for days 1–2, μg/L	1,167 (-484 to 2,820)	220 (103 to 337)	0.24
Mechanical ventilation	17	13	-
Length of stay, days	45.7 (27.8 to 63.6)	60.6 (27.9 to 93.4)	0.37

Data are mean (95% confidence interval) or number of patients. Early acute kidney injury (AKI) is defined as when creatinine reached the level for Risk within the first 7 days; late AKI occurred between days 8 and 60. Multiple organ failure is defined as 3 to 4 score points in two or more organ dimensions of the sequential organ failure assessment score. (Contingency table, Pearson chi-square test for categorical variables, and Student *t *test for continuous data.) MAP, mean arterial pressure.

**Table 5:** Early and late acute kidney injury: odds ratio for characteristics, multiple organ failure, and sepsis

	Early AKI	Late AKI		
			
Failure/Injury/Risk (all patients)	3/5/9 (17)	3/5/6 (14)	95% CI	OR
Age of <60/≥60 years	13/4	5/9	1.22 to 28.0	5.85
TBSA% of ≥50%/<50%	10/7	3/11	1.06 to 26.0	5.20
FTB% of ≥25%/<25%	12/5	4/10	1.26 to 28.5	6.00
Gender, male/female	12/5	9/5	0.29 to 6.04	1.33
Mortality (non-survivors)	6	5	0.22 to 4.30	0.98
Dialysis	3	1	0.26 to 30.27	2.79
MOF before AKI	13	10	0.26 to 6.52	1.30
Sepsis before AKI	11	8^a^	0.32 to 5.88	1.38
MOF and sepsis both before AKI	8	8	0.16 to 2.77	0.67
MAP episode <60 mm Hg on days 1–3	10	9	0.18 to 3.41	0.79
Adrenergic drugs on days 1–3	14	7	0.91 to 23.79	4.67

Data are number of patients. Early acute kidney injury (AKI) is defined as when creatinine reached the level for Risk within the first 7 days; late AKI occurred between days 8 and 60. Multiple organ failure (MOF) is defined as 3 to 4 score points in two or more organ dimensions of the sequential organ failure assessment score. Onset on the same day as AKI is included in the onset before category. ^a^Two more patients had sepsis before AKI, but there were 11 and 48 days, respectively, between their sepsis recordings and AKI onset, which was on days 25 and 60. Continuous variables were arbitrarily categorised: cutoff for age was 60 years, full thickness burn percentage (FTB%) was 25%, and percentage burned of total body surface area (TBSA%) was 50%. CI, confidence interval; MAP, mean arterial pressure; OR, odds ratio.

### Recovery and mortality

Renal function recovered completely during the time of admission to the burn unit among all patients who survived except for two: the dialysed patient who survived, whose renal function partially recovered, and one patient classified as Injury, who was transferred to another hospital before recovery. Mortality increased with increasing AKI class. The 11 patients having AKI and who died all had multiple organ failure. Among the four patients who required renal replacement therapy, plasma creatinine was within the reference range during the first day after injury in all cases but one, whose plasma creatinine was 126 μmol/L. All four had multiple organ failure before dialysis, and two had sepsis before. The remaining two already had SIRS on admission but no sepsis during their stay. The treatment with dialysis started on days 5 to 19 (10 to 15 days of treatment over 13 to 21 days), and the week-maximum plasma creatinine concentration and plasma urea before starting were 392.0 μmol/L (208.5 to 575.5) and 28.9 mmol/L (15.2 to 42.7), respectively. Two of the patients were oliguric the day before, and the patient with early dialysis (day 5) was oliguric 4 days before. Three of the dialysed patients died.

### Factors of importance in the development of acute kidney injury

#### Predisposing factors

Age, TBSA%, and extent of FTBs were greater among the patients who developed AKI (Table 2). We found no significant difference in these variables between the RIFLE classes when we analysed the AKI-classified patients, even if there was a trend toward increasing age (Table 3).

#### Sepsis

**Figure 2:**
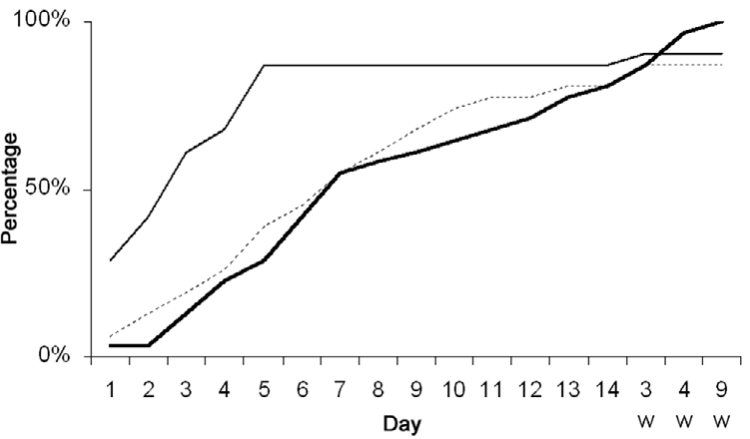
Day of onset of renal dysfunction, respiratory dysfunction, and sepsis. Cumulative percentage of the patients who developed renal dysfunction showing when their plasma creatinine concentration exceeded at least 1.5 × baseline (n = 31, thick line) and who developed severe respiratory dysfunction (sequential organ failure assessment score of 3 to 4 = PaO_2_/FiO_2 _[arterial partial pressure of oxygen/fraction of inspired oxygen] below 200 mm Hg, n = 28, thin line) and sepsis (n = 27, dotted line). X-axis shows the first 14 days after injury. The remaining times are weeks.

The patients who developed AKI (n = 31) all fulfilled the criteria for SIRS on day 1, and 87% (27 of 31) developed sepsis, of whom 19 were classified as severe sepsis or septic shock. Sepsis developed within a week before the first sign of renal dysfunction (reaching the level of Risk) in 48%, and most of these records of sepsis were classified as severe. Sepsis cumulative onset is presented in Figure 2. Sepsis also developed without inducing further renal dysfunction during the renal recovery period among seven patients.

#### Potentially nephrotoxic exposures

Twelve of the AKI-classified patients (39%) were treated with potentially nephrotoxic antibiotics and five of them required more than one. The total number of treatment periods among them was 25. In 6 of the 12 patients, an increase in the plasma creatinine concentration was seen after starting one treatment (starting day ranged from 3 to 92 after the burn) and severe sepsis was present on all of these occasions.

Seven of the patients who did not develop AKI were exposed to intravenous contrast (CT scans). One of the patients who were classified as Failure was exposed on day 2 in parallel with increasing plasma creatinine concentration, and 2 patients classified as Risk were exposed 6 and 19 days, respectively, before the increase. Plasma myoglobin (highest value days 1 to 2) values were 1,606 μg/L (95% CI 677 to 2,534) in the non-AKI group and 712 μg/L (95% CI -111 to 1,537) among the patients who developed AKI (*P *= 0.22).

#### Relation to other organs

**Figure 3:**
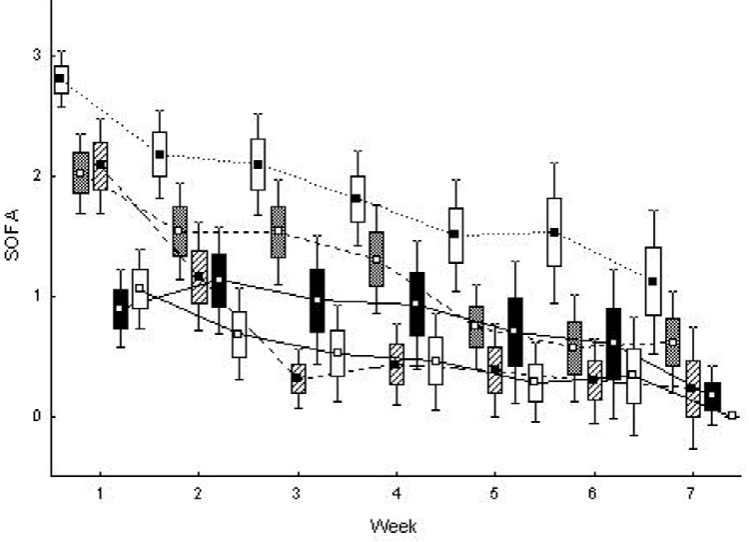
Maximum sequential organ failure assessment (SOFA) score among the patients who developed renal dysfunction (n = 31). SOFA score is calculated on the maximum value for each of five organ dimensions weekly during the first 7 weeks after injury: maximum SOFA respiratory dimension (closed square, open box), cardiovascular dimension (open square, shaded box), coagulation dimension (closed square, diagonal pattern in the box), renal dimension (open square, closed box), and hepatic dimension (open square, open box). Squares indicate the mean, the box indicates standard error, and whiskers indicate 95% confidence interval.

Organ dysfunction in general was most pronounced during the first weeks after injury (Figure 3), and outcome depended on the number and degree of failing organs. Maximum SOFA total was 14.1 (95% CI 12.5 to 15.6) among non-survivors with AKI compared with 10.2 (95% CI 9.0 to 11.4) among survivors with AKI (*P *<0.001, adjusted for age and TBSA% *P *= 0.001). But when each dimension was analysed among the 31 patients with AKI, only the renal and cardiovascular dimensions were higher among the patients who died (maximum renal dimension was 2.7 (95% CI 1.9 to 3.6) and the cardiovascular dimension was 3.6 (95% CI 3.3 to 4.0) among non-survivors compared with 1.2 (95% CI 0.7 to 1.6) and 2.4 (95% CI 2.1 to 2.7) among survivors (*P *<0.001 for both dimensions, adjusted for age and TBSA% *P *= 0.002 for renal dimension, and *P *<0.001 for cardiovascular dimension)). Pulmonary dysfunction preceded AKI, and 97% (30 of 31) required mechanical ventilation and 61% (19 of 31) had a PaO_2_/FiO_2 _ratio reduced to less than 200 mm Hg (scoring 3 to 4 points on the SOFA respiratory dimension) within the first 3 days.

## Discussion

This is, to our knowledge, the first study to assess AKI in burns using a prospective protocol with the RIFLE classification as well as the assessment of organ failure, SIRS, and sepsis using conventional criteria and definitions. The study adds new and important information about several topics in a comprehensive way. It shows that AKI is common, that AKI corresponds to about a quarter of those with a TBSA% of more than 20%, and that survivors can recover from AKI. AKI also develops soon after injury and is closely paralleled by dysfunction of other organs. We found that AKI was preceded by lung dysfunction in almost all of the cases, as has previously been claimed by Sheridan and colleagues [14]. Only the cardiovascular dysfunction (maximum SOFA) and AKI were associated with mortality. The relation with sepsis was not as incontestable as is usually claimed since sepsis was not always followed by renal dysfunction.

Renal dysfunction seems to follow a course similar to that of other dysfunctioning or failing organs, more so as the time delay between different organ dysfunctions can be considered (at least to some extent) as being marker-specific rather than organ-specific. The actual impact on the kidneys is likely to happen before the increase in plasma creatinine concentration is detected. For example, Kang and colleagues [35] found raised 24-hour urinary *N*-acetyl-beta-D-glucosaminidase activity (a marker of proximal tubular dysfunction or damage) on day 1 among the 12 burned patients whom they studied (30% TBSA%). It was almost doubled on day 1, continued to increase, and peaked on day 7.

### Incidence and occurrence

We found AKI to be common, with an incidence of about a quarter of major burns, which is similar to that reported in a recent paper by Coca and colleagues [19] and slightly less than reported in a letter from Lopes and colleagues [20]. Median time to reaching respective RIFLE class was 10 days in our AKI group, which is the same as that reported by Lopes and colleagues [20]. However, unlike Coca and colleagues [19], we did not find a difference in the time of occurrence between RIFLE classes. The requirement of renal replacement therapy among patients with burns who require intensive care seems not to differ from that of general intensive care units (ICUs). The percentage of renal replacement therapy in our study (3.1%) is close to that reported in ICU patients (Hoste and colleagues [2] 4.1%, Bell and colleagues [36] 2.5%, Uchino and colleagues [4] 4.3%, and Dalfino and colleagues [37] 8.1%) and in most studies of patients with burns (Table 1).

### Recovery or mortality

All of the surviving patients in the present study recovered renal function (defined according to RIFLE). This is consistent with findings reported by several others [8,10,12,15]. In a multicentre long-term follow-up of patients in intensive care who had required renal replacement while they were in hospital, 3.4% (34 of 998) of those who survived developed late end-stage kidney disease, as identified from a nationwide register for chronic renal disease [38].

Mortality increased with increasing RIFLE class, and in the studies of Coca and colleagues [19] and Lopes and colleagues [20] mortality rates were 60% and 75%, respectively, in the Failure class, whereas the rate was 5 of 6 in our study. Overall mortality rates were 14% in our study, 13% in the study of Coca and colleagues, and 18% in the study of Lopes and colleagues. ICU mortality among RIFLE-Failure-classified patients seems to be somewhat lower than among burned patients who were classified as Failure. Hoste and colleagues [2] reported 26% mortality in the Failure class from a study of critically ill patients, and Lopes and colleagues [39] found a 55% mortality in the Failure class among patients with sepsis.

### Pathophysiology of renal dysfunction in burns

The reason for AKI among patients with major burns may be multifactorial. We found that the acute increase in plasma creatinine concentration was preceded by the initial inflammatory response (SIRS) and pulmonary dysfunction. Pulmonary dysfunction after trauma has been suggested to promote pathogenic inflammation and the development of multiple organ failure, including renal failure [40]. In our recent study of acute respiratory dysfunction in patients with major burns, we noted that acute respiratory distress syndrome occurs soon after the burn – usually within 3 days – and that renal dysfunction was more common among the patients with the most severe respiratory dysfunction [32]. This, together with the early onset of organ dysfunction, including renal dysfunction, suggests that it is the burn and resuscitation rather than infective complications that are responsible for the failing organs.

### Sepsis

Sepsis occurred in 87% of the AKI group, which is of the same magnitude as reported in previous burn studies [11,19]. We found that severe sepsis was associated with AKI, even if not all episodes of severe sepsis caused renal dysfunction. In four of six cases in which AKI was of latest onset (days 18 to 60 after burn), it was not preceded by sepsis, contradicting the idea that AKI of late onset was associated mainly with sepsis [15,17]. Chrysopoulo and colleagues [16] found that AKI among survivors was not the result of sepsis since it preceded sepsis in their study. Another interesting finding is that we found sepsis during the renal recovery period without inducing further renal dysfunction, which has not been previously reported. This finding indicates that at least some of the time-associated episodes of sepsis and renal dysfunction may also be just time-related rather than the result of cause and effect – a possibility that is usually not discussed in studies of burned patients where AKI is considered to be strongly associated with sepsis [10,12,15,17,19].

### Predisposing factors

We found age, TBSA%, and FTB% to be predisposing factors for AKI but were unable to show the corresponding relation for severity of AKI, most probably because of a lack of power. Coca and colleagues [19] also found older patients in the AKI group, whereas others (for example, Holm and colleagues [11] and Kim and colleagues [17]) found a higher TBSA% in the AKI group, but not advanced age. In the study by Kim and colleagues, mean TBSA% was unusually high in the AKI group (80%) whereas age was relatively young (42 years).

### Method

It is important to evaluate the characteristics of patients with burns. Effects are seen on incidence of organ dysfunction and on outcome by the number of patients who have treatment withheld or withdrawn. In different studies, the size of this group has been in the range of 5% to 11% [6,14]. In a number of studies, no such data are presented [8-13,15-17,19-21]. We have excluded all patients who died within the first 48 hours, including cases of initial withholding or withdrawal of treatment. The exclusion criteria of 'short hospital stay' has been used by others [7].

The potential selection bias from excluding the patients with the worst (death within 2 days) and the best (short duration of stay) outcomes has probably influenced the incidence of AKI in this study. The finding that young age is a risk factor for early AKI can also be explained by this selection bias since older patients with extensive burns more often have a lethal outcome.

The fluid resuscitation early after burn is a problem when using the RIFLE criteria and not having a true baseline plasma creatinine concentration taken. The initially low concentrations in plasma, however, should be of the same magnitude among burn patients as a group, reflecting a physiological response to the burn and the fluid resuscitation. Hence, using the RIFLE classification may still be reliable for comparing incidences of acute renal dysfunction between studies of burn patients. The same 'misclassification' problem is, however, likely to occur among other patient groups who are subjected to aggressive fluid resuscitation (ICU patients with major trauma or those with severe sepsis) and whose true baseline may be unknown. Whether the RIFLE should be modified for these circumstances needs to be further examined.

## Conclusion

AKI is common, develops soon after the burn, and is paralleled by multiple organ dysfunction or failure, which also appear early. Among the dysfunctioning organs, cardiovascular dysfunction (SOFA) together with AKI was associated with a higher mortality. The prognosis for minor dysfunction remains good and survivors recover from AKI, whereas renal failure still carries a high mortality. Pulmonary dysfunction preceded AKI and 30 of the 31 patients with AKI required mechanical ventilation whereas only half of those with no AKI required mechanical ventilation. Sepsis was not always followed by AKI.

## Key messages

• Acute kidney injury (AKI) is common, develops soon after the burn, and is paralleled by multiple organ dysfunction.

• Cardiovascular dysfunction together with AKI was associated with a higher mortality.

• The prognosis for minor dysfunction remains good and survivors recover from AKI, whereas renal failure still carries a high mortality.

## Abbreviations

AKI: acute kidney injury; BW: body weight; CI: confidence interval; CT: computed tomography; FiO_2_: fraction of inspired oxygen; FTB: full thickness burn; ICU: intensive care unit; PaO_2_: arterial partial pressure of oxygen; RIFLE: Risk, Injury, Failure, Loss of kidney function, and End-stage kidney disease; SIRS: systemic inflammatory response syndrome; SOFA: sequential organ failure assessment; TBSA%: percentage burned of total body surface area.

## Competing interests

The authors declare that they have no competing interests.

## Authors' contributions

IS participated in the design of the study, acquired the data and performed the statistical analysis, participated in the interpretation of data, and drafted the manuscript. ZB critically revised the study. FS had the original idea and participated in the design of the study, interpretation of data, and drafting of the manuscript. All authors read and approved the final manuscript.
